# A systematic review of the epidemiology of Hepatitis E virus infection in South – Eastern Asia

**DOI:** 10.1080/21505594.2020.1865716

**Published:** 2020-12-29

**Authors:** Yakubu Egigogo Raji, Ooi Peck Toung, Niazlin Mohd Taib, Zamberi Bin Sekawi

**Affiliations:** a Department of Medical Microbiology and Parasitology, Universiti Putra Malaysia 1, Malaysia; b Department of Pathology, Clinical Microbiology Unit College of Health Sciences Ibrahim Badamasi Babangida University Lapai Nigeria, Nigeria; c Department of Veterinary Clinical Studies Faculty of Veterinary Medicine, Universiti Putra Malaysia 2, Malaysia

**Keywords:** Hepatitis E, Hepatitis E virus, seroprevalence, sporadic HEV infection, HEV outbreaks, South – eastern Asia

## Abstract

Hepatitis E virus (HEV) infection is an emerging zoonotic viral disease, with an increasingly international public health challenge. Despite the concerns that the global disease burden may be underestimated. Therefore, evaluation of the disease epidemiology in South – eastern Asia through a systematic review will assist in unraveling the burden of the disease in the subregion. A priori protocol was prepared for the systematic review and followed by a literature search involving five electronic databases. Identified publications were screened for high quality studies and the elimination of bias and relevant data extracted. A total of 4157 citations were captured, and only 35 were included in the review. A wide range of HEV seroprevalence was recorded from 2% (urban blood donors in Malaysia) to 77.7% (lowland communities in Lao PDR). Sporadic HEV infection and epidemics were also detected in the subregion. Indicating hyperendemicity of the disease in South – eastern Asia.

## Introduction

Hepatitis E virus (HEV) is the viral agent that causes an infectious disease known as hepatitis E. Hepatitis E to a large extent, manifests as acute icteric hepatitis, otherwise known as acute viral hepatitis (AVH) [1]. AVH is usually asymptomatic or self – limiting, but it may progress to acute liver failure (ALF) in some individuals. ALF is often associated with increased disease severity with extrahepatic manifestations and mortality [2]. Additionally, HEV infection may persist, leading to chronicity in immunocompromised persons. The disease severity and mortality are often higher in chronic infection and pregnant women with AVH. Hepatitis E mortality in the general population is usually between 0.2% – 4%. On the other hand, mortality ranges between 15% – 100% in pregnant women [3,4] and patients with chronic liver disease [5].

Although the actual burden of hepatitis E is not known, yet it is considered an emerging disease with an increasing public health threat globally [6]. Despite underestimating, the global burden of HEV infection is estimated at 2.3 billion, with an annual global incidence of 20 million [7,8]. HEV is responsible for both sporadic and epidemic infections. HEV is regarded as the commonest cause of acute viral hepatitis in the world [8]. HEV epidemics have been reported only in the developing countries of Asia, Africa, and in Mexico [9].

HEV is a small spherical virus with a diameter of 27–34 nanometers [10]. It is a non – enveloped positive – sense, single – stranded RNA virus with an approximate 7.2Kb genome [11,12]. The genome of HEV has three open reading frames (ORF); ORF1, ORF2, and ORF3. HEV belongs to the *Hepeviridae* family and has two genera; the *Orthohepevirus* (strains infect mammals and birds) and *Piscihepevirus* (strains infect fish) [10]. The genus *Orthohepevirus* has four distinct species (A, B, C, and D) while the second genus has one species; *Piscihepevirus A*. The *Orthohepevirus A* has eight known genotypes named HEV – 1 to HEV – 8 [13]. HEV – 1 and HEV – 2 are exclusively human viruses and enterically transmitted. Thus, responsible for most of the infections in developing countries. HEV – 1 and HEV – 2 are also associated with epidemics and severe infection in pregnancy [2]. Thus, often referred to as the “epidemic genotypes”. On the other hand, HEV – 3 and HEV – 4 are swine genotypes that cause zoonotic infection in humans [9]. HEV – 3 and HEV – 4 are mostly responsible for infections in the developed countries [14] and for chronic HEV infection [2]. Also, HEV – 7 [15] and Orthohepevirus C [16] have been reported as causes of chronic HEV infection in humans.

Systematic reviews (SR) are essential tools in human medical research, thus crucial for investigating HEV seroprevalence. SR of HEV epidemiology have been conducted in Africa [17,18], Europe as well as in some selected high – income countries [19]. However, there is no comprehensive SR of HEV infection in South – eastern Asia (SEA). SEA is a unique region; the region is multicultural, multiracial, multireligious, and comprises of countries that have both low and high income. These factors affect the distribution pattern of HEV; from outbreaks, sporadic infections, prevalent genotypes, mode of transmission to at risk population. Thus, a better understanding of hepatitis E epidemiology will provide more details on the pattern of the disease distribution in this region. It will also assist in implementing informed policy decisions and evidence based control measures for hepatitis E and associated healthcare challenges.

Therefore, the purpose of this study was to evaluate the epidemiology of HEV infection in SEA by reviewing and summarizing pertinent peer – reviewed publications. Sub – objectives were to determine the hepatitis E disease rates (seroprevalence, sporadic infection, outbreaks) in the subregion. The study also assessed the mode of disease transmission and identified circulating HEV genotypes. Additionally, the authors identified knowledge gaps and made recommendations for improved HEV studies. Authors also made recommendations for governments to implement measures to prevent HEV infection in at – risk populations and in the general population in the SEA subregion.

## Methods

### Scope

A priori protocol (S1 File) was prepared based on the Preferred Reporting Items for Systematic Reviews and Meta – Analysis (PRISMA) guidelines [20] using the PRISMA assessment checklist (S2 File), before conducting this SR.

For this SR, the eligibility (inclusion and exclusion) criteria were defined using the acronym PICOT:

- **Population**: Inclusion; studies conducted in the population of the South – eastern Asia countries (study location) as outlined by the United Nations [21] were included in the study. The details of the list of these countries are given in S3 File. Studies that involved susceptible groups (pregnant women, patients with co – infection, patients with chronic disease, animal farm owners, farm workers, veterinary officers, displaced persons, prisoners, homeless, sex workers, illicit drug users, rural dwellers) and non – susceptible groups (healthy general population, healthy blood donors, urban residents) and participants of all age range were included.

Exclusion; studies on the populations outside the South – eastern Asia countries, animal studies, environmental studies, and studies without a clear population description were considered irrelevant for this review.

- **Intervention (Exposure**): Inclusion; studies that measured the total HEV antibodies (IgG and IgM), IgG only, IgM only, and HEV RNA for the circulating virus genotypes were deemed relevant for this SR.

Exclusion; studies without a clear description of serological assays used were excluded from this study.

- **Context**: Inclusion; this study included all observational (cross – sectional studies, intervention studies, cohort studies, case – control studies, case series, longitudinal prevalence studies, seroprevalence studies, prevalence surveys) studies that report the prevalence of HEV infection in the study location with English as the language of publication.

Exclusion; studies published in other languages other than English, studies that did not separate the prevalence of HEV from other viral diseases, studies covering topics other than HEV epidemiology (laboratory studies on the pathogenesis of diseases, molecular biology), and animal studies only were excluded. Also excluded were studies on case reports, letters, books, dissertations, review articles, unpublished reports, and conference papers.

- **Outcome**: Patients with serological evidence of HEV exposure (from seroprevalence, outbreaks, sporadic cases), risk factors, mode of transmission, and circulating genotypes.
- **Time frame**: No limitations were placed on the year of publication.

### Search strategy

The electronic search was conducted on 22 March 2020. The strategy embraced the assessment of all relevant literature citations captured by applying the search algorithm in five electronic bibliographic databases (Scopus, Science Direct, PubMed, MEDLINE, and ASEAN Citation Index). Also, a gray literature search was conducted via hand searching references of selected (review) articles and conference proceedings. Additionally, a related internet search was done in Google Scholar and Google on 10 June 2020. Details of specific algorithms used for searching each of the databases are outlined in the study protocol (S1 File). However, a sample search algorithm is given as follows; (“Hepatitis E Virus” OR “HEV infection” OR “HEV” OR “Viral Hepatitis E” OR “Hepatitis E” OR “Hepatitis E virus infection” OR “Hepatitis E antibodies”) AND (Seroepidem* OR “Prevalence” OR Epidem* OR “Survey” OR “Seroprevalence”) AND (“Indonesia” OR “Vietnam” OR “Thailand” OR “Singapore” OR “Malaysia” OR “Philippines” OR “Cambodia” OR “Myanmar” OR “Burma” OR “Laos” OR “Brunei” OR “Timor-Leste”).

### Data management

The obtained searched articles were compiled and de-duplicated in an MS Excel spreadsheet. All steps of the SR, from screening to data extraction, were carried out on an Excel spreadsheet. The final dataset on the MS Excel spreadsheet was then subjected to further analysis.

**Selection process; t**he study selection was conducted by two independent reviewers, and a third reviewer decided about uncertainties based on discussion and consensus.

**Data collection process; e**xtraction of data was conducted simultaneously with the full text searching. Relevant information were extracted from each article included and recorded immediately in the respective data extraction files. Two independent reviewers carried out this process, and two others checked the information for verification.

### Quality assessment

Each article included in this SR was deemed relevant after meeting the inclusion and exclusion criteria. The quality of each article was then evaluated based on the prevalence critical appraisal instrument developed by [22] for seroprevalence studies and the critical appraisal checklist by [23] for case series and outbreak studies. The ten questions used in each of the critical appraisal instrument was answered either with Yes, No, Unclear, or not applicable. Articles with ≤60% score, or ≥3 U, were considered to have failed the quality assessment test and were not included in the study. The details of the quality assessment and the articles assessed are given in the S1 Table.

## Results

**Figure 1.**
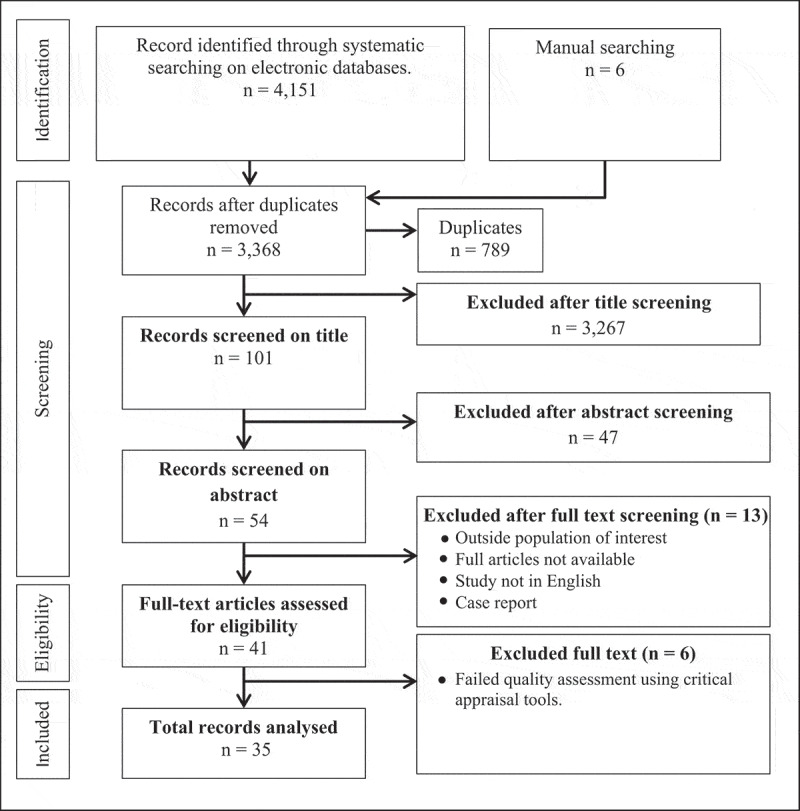
Flow diagram

**Figure 2.**
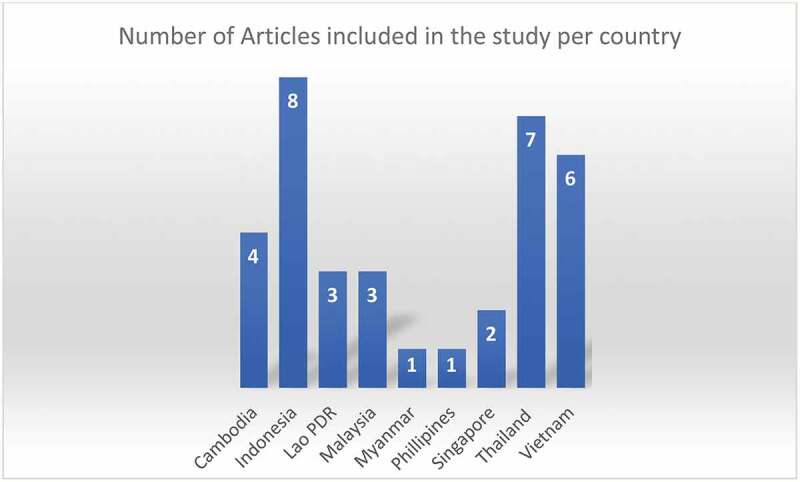
Country distribution of number of articles included in the study

The systematic search conducted on the electronic databases captured 4151 citations, and additional six citations were found by manual searching. After a series of screenings, as shown in Figure 1 [20], 41 articles met the eligibility criteria, and a list of the articles is given in the S4 file. After quality assessment, 35 articles from 9 out of the 11 SEA countries were included. The distribution of included publication according to country is given in Figure 2. At the same time, the descriptive characteristics of the included studies are given in Table 1.

**Table 1.** Descriptive characteristics of included studies

						Outcome			
S/N	Author	Country	Year of Publication	Study Design	Seroprevalence	Sporadic infection	Outbreak investigation	Genotype study	Ref
1	Nouhin et al.,	Cambodia	2015	Co-S	Yes	Yes	No	No	[61]
2	Yamada et al.,	Cambodia	2015	Cr-S	Yes	No	No	Yes	[62]
3	Nouhin et al.,	Cambodia	2016	Pr-S	Yes	No	No	Yes	[63]
4	Nouhin et al.,	Cambodia	2019	Ep-S	Yes	No	No	No	[57]
5	Sedyaningsih-Mamahit et al.,	Indonesia	2002	Ot-I	No	Yes	Yes	No	[64]
6	Corwin et al.,	Indonesia	1995	Cr-S	Yes	No	No	No	[48]
7	Surya et al.,	Indonesia	2005	Se-S	Yes	No	No	No	[24]
8	Utsumi et al.,	Indonesia	2011	Pr-S	Yes	No	No	No	[25]
9	Wibawa et al.,	Indonesia	2004	Pr-S	Yes	No	No	No	[26]
10	Wibawa et al.,	Indonesia	2007	Pr-S	Yes	Yes	No	Yes	[27]
11	Widasari et al.,	Indonesia	2013	Pr-S	Yes	No	No	No	[71]
12	Achwan et al.,	Indonesia	2007	Pr-S	Yes	No	No	No	[28]
13	Khounvisith et al.,	Lao PDR	2018	Cr-S	Yes	No	No	No	[29]
14	Tritz et al.,	Lao PDR	2018	Ser-S	Yes	No	No	No	[30]
15	Holt et al.,	Lao PDR	2016	Cr-S	Yes	No	No	No	[49]
16	Ng et al.,	Malaysia	2000	Ser-S	Yes	No	No	No	[31]
17	Seow et al.,	Malaysia	1999	Pr-S	Yes	No	No	No	[50]
18	Hudu et al.,	Malaysia	2018	Mo-Ep	Yes	No	No	Yes	[32]
19	Uchida et al.,	Myanmar	1993	Ep-S	No	No	Yes	No	[70]
20	Gloriana-Barzaga et al.,	Philippines	1997	Ca-S	No	Yes	No	No	[33]
21	Chow et al.,	Singapore	1996	Ser-S	Yes	No	No	No	[60]
22	Wong et al.,	Singapore	2019	Sp-S	Yes	Yes	No	Yes	[58]
23	Hinjoy et al.,	Thailand	2013	Cr-S	Yes	No	No	No	[34]
24	Sa-nguanmoo et al.,	Thailand	2015	Cr-S	Yes	No	No	No	[35]
25	Poovorawan et al.,	Thailand	1996	Pr-S	Yes	Yes	No	No	[36]
26	Siripanyaphinyo et al.,	Thailand	2014	Ca-S	Yes	Yes	No	Yes	[37]
27	Pilakasiri et al.,	Thailand	2009	Sr-S	Yes	No	No	No	[38]
28	Jupattanasin et al.,	Thailand	2019	Sr-S	Yes	No	No	No	[39]
29	Gonwong et al.,	Thailand	2014	Ser-S	Yes	No	No	No	[40]
30	Tran et al.,	Vietnam	2003	Mo-Ep	Yes	No	No	No	[41]
31	Hau et al.,	Vietnam	1999	Cr-S	Yes	No	No	No	[42]
32	Hoan et al.,	Vietnam	2019	Cr-S	Yes	No	No	No	[43]
33	Hoan et al.,	Vietnam	2015	Cr-S	Yes	No	No	Yes	[44]
34	Berto et al.,	Vietnam	2018	Co-S	Yes	No	No	No	[45]
35	Corwin et al.,	Vietnam	1996	Cs-C	Yes	Yes	No	No	[46]

Co-S; cohort study, Cr-S; cross-sectional study, Cs-co; case-control study, Ep-S; Epidemic study, Mo-Ep; molecular epidemiologic study, Ot-I; outbreak investigation study, PDR; peoples’ democratic republic, Pr-S; prevalence study, Ser-S; seroprevalence study, Se-S; serological survey, Sp-S; seroepidemiologic study, Sr-S; serosurvey

### Anti-HEV antibodies seroprevalence

**Figure 3.**
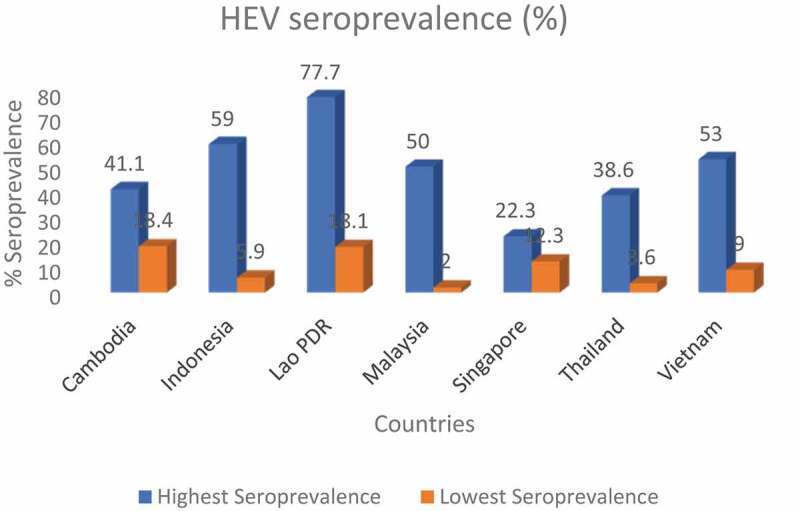
Cross-section of % seroprevalence across the countries

Data on HEV antibodies seroprevalence was extracted from 33 studies in seven SEA countries. A summary of the major characteristics of the studies, including country, year of sampling, sample demographics, and assay used, is presented in Table 2. The distribution of the studies for each country; Cambodia (n = 3), Indonesia (n = 8), Lao PDR (n = 3), Malaysia (n = 3), Singapore (n = 2), Thailand (n = 7) and Vietnam (n = 6). Included studies used a wide range of assays for the anti-HEV antibody seroprevalence. However, the predominant assay employed is the kit from MP Biomedical, previously called Genelabs (n = 9). MP Biomedical is followed by the Wantai Bio-Pharm (n = 6). The variation seroprevalence across the countries is summarized in Figure 3.

**Table 2.** Anti-HEV antibodies seroprevalence in Southeast Asia

S/N	Country	% seroprevalence	Sample demographics	Sex	Sample size	Year of sampling	Diagnostic Method	Assay type used	Source
1	Cambodia	41.1	Preserved human plasma samples, (median 37) 18–91 years old	M/F	2004	1996–2007	Total Ig	Wantai Bio-Pharm	Nouhin et al., 2019
2		28.2	Healthy adult blood donors, (median 29) 24–34 years old	M/F	301	2014	IgG	Wantai Bio-Pharm	Nouhin et al., 2016
3		18.4	General population, (30.5 ± 18.8) 7–90 years old	M/F	868	2010–2014	Total Ig	Institute of immunology Co	Yamada et al., 2015
4		30.1	Preserved samples of patients with unexplained febrile illness and liver enzymes elevations, 0–59 years old	M/F	825	2008–2010, 2013	Total Ig	Wantai Bio-Pharm	Nouhin et al., 2015
5	Indonesia	6.5 (Bali; 20, Lombok; 17, Surabaya; 0.5)	Healthy individuals and voluntary blood donors from three different regions, 16–64 years old	M/F	1,115	1996	IgG	Mizuo et al., method	Wibawa et al., 2004
6		18	Voluntary blood donors from Bali region, 16–64 years old	M/F	797	2003	IgG	Mizuo et al., method	Wibawa et al., 2004
7		9.9 (Java; 3.7, Bali; 11.6)	Healthy individuals and swine farm workers in Java and Bali communities,	M/F	253	2008–2010	IgG	EIA Institute of immunology, Tokyo	Utsumi et al., 2011
8		18	Pregnant women in Bali, (27 ± 5) 16–45 years old	F	819	2003	IgG	Mizuo et al., method	Surya et al., 2005
9		40.4	Acute hepatitis patients, (31.1 ± 11.9) 12–62	M/F	57	2003–2006	IgG	Mizuo et al., method	Wibawa et al., 2007
10		59	Patients with previous history of HEV infection from previous outbreak and control subjects, (29.9 ± 16.6) 2–80 years old	M/F	445	1993	IgG	Genelabs Diagnostics & Western blotting	Corwin et al., 1995
11		Java; 5.1, Bali; 11.6	Swine farm workers and local residents in Java and Bali,	M/F	490 (Java; 291, Bali; 199)	2011	Total Ig	MPD HEV ELISA 4.0 v; MP Biomedicals	Widasari et al., 2013
12		5.9	General population, 1–61 years	M/F	581	2005	IgG	In-house assay	Achwan et al., 2007
13	Lao PDR	51.8 (risk group; 59.1, control group; 43.9)	Healthy villagers, (mean 48) 18–85 years old	M/F	326 (risk group; 171, control group; 155)	2016	Total Ig	AB diagnostics	Tritz et al., 2018
14		Risk group; 41, Control group; 18.1	Professionals exposed to Pigs & Blood donors’ control, 15 – >50 years old	M/F	349 (risk group; 139, control group; 210)	2015	IgG	Euroimmum, Lubeck	Khounvisith et al., 2018
15		Upland; 48.6, lowland; 77.7	Human population in upland and lowland communities,	M/F	870	2011	Total Ig	MP Diagnostics	Holt et al., 2016
16	Malaysia	9.8	Chronic hepatitis B patients, (<30 – ≥50) years old	M/F	82	2015–2016	Total Ig	Wantai Bio-Pharm	Hudu et al., 2018
17		26.7 (Blood donors; 2, Betau; 44, Parit Tg; 50)	Urban blood donors and healthy individuals in two Aboriginal communities, 1–80 years old	M/F	232	1990, 1998	IgG	AMRAD Biotech	Seow et al., 1999
18		14	Human immunodeficiency Virus Type 1 infected subjects, (<20 – ≥40) years old	M/F	145	2000	Total Ig	Abbott Laboratory & Genelabs Diagnostics	Ng et ai., 2000
19	Singapore	22.3	Anonymized residual human serum samples	M/F	3261	2007–2016	IgG	MP Diagnostics & Mikrogen Kits	Wong et al., 2019
20		12.3 (healthy group; 10.5, patients’ group; 14.7)	Healthy general population and liver disease patients, (mean 51) 14–95 years old	M/F	219 (healthy group; 124, patients’ group; 95)	1993	IgG	Genelab	Chow et al., 1996
21	Thailand	37	Healthy individuals, (0–69 years old	M/F	721	2014	IgG	Euroimmum, Lubeck	Sa-nguanmoo et al., 2015
22		11.5	Nursing Army cadets, (20 ± 3.6) 16–41 years old	F	381	2009	IgG	WRAIR EIA	Pilakasiri et al., 2009
23		23	Pig farmers and those without exposure to pigs, 15 – > 65 years old	M/F	513	2010–2011	Total Ig	WRAIR EIA	Hinjoy et al., 2013
24		29.7	Archived serum samples of healthy adult blood donors, (median 38) 18–64 years old	M/F	630	2013	IgG	Euroimmum, Lubeck	Jupattanasin et al., 2019
25		Blood donors; 15.7, pregnant women; 9, children; 3.6	Adult blood donors, pregnant women and children, 1 – >50 years old	M/F	900	1992–1994	IgG	Genelabs Diagnostics	Poovorawan et al., 1996
26		14	Men of Royal Thai Army recruits, 18–30 years old	M	7760	2007–2008	IgG	DIA. PRO	Gonwong et al., 2014
27		38.6	Acute hepatitis patients, 1–90 years old	M/F	548	2008–2009, 2011	IgG	DIA.PRO Diagnostic	Siripanyaphinyo et al., 2014
28	Vietnam	Healthy; 31, exposed; 53	Healthy individuals and those exposed to pigs, (median 41) 18–78 years old	M/F	451 (healthy; 168, exposed; 283)	2016–2017	Total Ig	MP Biomedicals	Hoan et al., 2019
29		Patients; 45, control; 31	Patients with hepatitis E infection and healthy population control, 9–84 years old	M/F	1658 (patients; 1318, control; 340)	2012–2013	Total Ig	MP Biomedicals	Hoan et al., 2015
30		Famer cohort; 16, control; 31.7	Farmer cohort and Hospital population Controls,	M/F	2007 (farmers; 281, control; 1726)	2009–2014	IgG	Wantai Bio-Pharm	Berto et al., 2018
31		9	Riverine dwellers, (20.45 ± 20.36) 0–87 years old	M/F	646	1994	IgG	Abbott laboratories	Hau et al., 1999
32		42	Liver disease patients and healthy persons, 21 – >61 years old	M/F	185	1998–2001	Total Ig	-	Tran et al., 2003
33		Cases; 21, control 14	Acute hepatitis patients, (26 ± 11) 1–68 years old	M/F	375 (cases; 188, control; 187)	1993–1995	IgG	Abbott laboratories	Corwin et al., 1996

F; female, Ig; immunoglobulin, IgG; immunoglobulin G, IgM; immunoglobulin M, M; male, M/F; male/female

**Figure 4.**
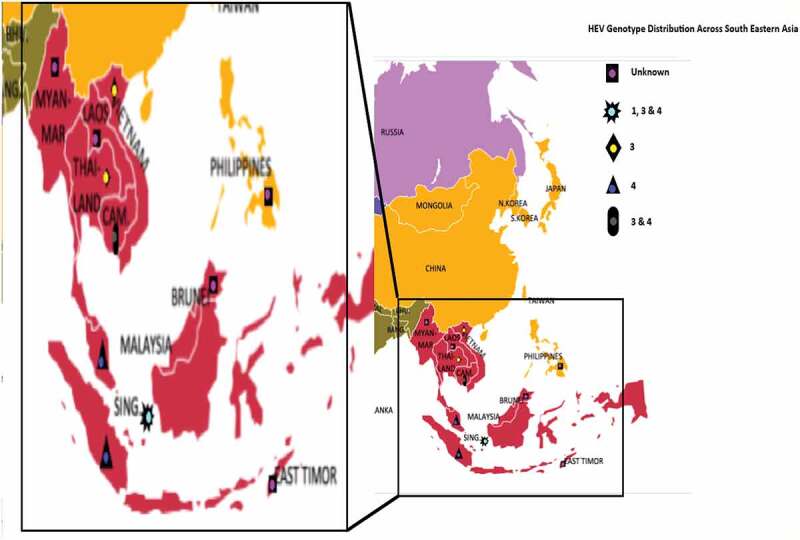
Map of South Eastern Asia showing the HEV genotype distribution in the region. Brunei, Laos, Myanmar, Phillipines, and Timor-Leste have unknown HEV genotype (data not available). In Singapore HEV genotypes 1, 3 and 4 are present. Thailand and Vietnam both have HEV genotype 3. HEV genotype 4 is prevalent in Indonesia and Malaysia. While Cambodia have both HEV genotypes 3 and 4

### Acute sporadic HEV infection

Data on sporadic HEV infection was captured from acute (sporadic) hepatitis cases in eight studies from six countries (Cambodia: n = 1, Indonesia: n = 2, Philippines: n = 1, Singapore: n = 1, Thailand; n = 2, and Vietnam: n = 1). Table 3 presents the characteristics of the studies, including country, year of sampling, method of diagnosis, case demographics, total sampled patients, number of cases, and percentage seropositivity.

**Table 3.** HEV Sporadic infections in South East Asia

S/N	Country	Year of sampling	Method used in Diagnosis	Case demographics	Toatal Sampled Patients	Number of cases	% seropositivity	Source
1	Cambodia	2008–2010, 2013	IgM	Preserved samples of patients with unexplained febrile illness and liver enzymes elevations, 0–59 years old	825	9	1.1	Nouhin et al., 2015
2	Indonesia	1997–1998	IgM/RT-PCR	Acute hepatitis patients, (32 ± 15.1) 1–70 years old	182	160	88	Sedyaningsih-Mamahit et al., 2002
3	Indonesia	2003–2006	IgM/RT-PCR	Acute hepatitis patients, (31.1 ± 11.9) 12–62 years old	57	2	3.5	Wibawa et al., 2007
4	Philippines	1992	IgM	Viral hepatitis patients, (31 SD 16.62)	65	4	6.15	Gloriani-Barzaga et al., 1997
5	Singapore	2012–2016	IgM	Acute hepatitis patients, 0–65+ years old	5080	503	10	Wong et al., 2019
6	Thailand	1992–1994	IgM	Acute viral hepatitis, 16–33 years old	68	5	7.4	Poovorawan et al., 1996
7	Thailand	2008–9, 11	IgM/RT-PCR	Acute hepatitis patients, 1–90 years old	614	26	4.2	Siripanyaphinyo et al., 2014
8	Vietnam	1993–1995	IgM	Acute hepatitis patients, (26 ± 11) 1–68 years old	188	6	3.2	Corwin et al., 1996

### HEV outbreaks

HEV outbreaks were recorded in two out of the 11 SEA countries studied. The outbreaks were reported in two studies, and details are summarized in Table 4.

**Table 4.** HEV outbreak infections in South East Asia

Country	Year	Suspected cases	Confirmed cases	%	Case fatality rate	Most affected population	Clinical attack rate	Commonest Presenting symptom	Source
Myanmar	1989	160	108	67.5 (admitted cases; 83.7, not admitted; 30.6)	NR	-	NR	Jaundice	Uchida et al., 1993
Indonesia	1997–1998	235	110	46.8	0	Female	19%	Dark urine	Sedyaningsih-Mamahit et al., 2002

NR; not reported,

### Risk factors and Mode of transmission

Risk factors, at risk groups, and the mode of HEV transmission was assessed and summarized in Table 5.

**Table 5.** At risk groups, risk factors and transmission route for HEV infection

Source	Country	Transmission route	Risk	factor/	At risk group		
			Risk practice/condition	Gender	Age	Occupation	Residence
Yamada et al., 2015	Cambodia	Blood-borne	Blood transfusion	Male	Older age	House workers	NR
Nouhin et al., 2015	Cambodia	NR	NR	Male	50–59 years old	NR	NR
Nouhin et al., 2016	Cambodia	Blood-borne, water-borne	Blood transfusion, Drinking fecally contaminated water	NR	Age 40 years and above	NR	NR
Nouhin et al., 2019	Cambodia	NR	NR	Male	Age 30 years and above	NR	Urban
Corwin et al., 1995	Indonesia	Water-borne	Poor water-related sanitary/hygienic practice, dependence on a single water source, and subnormal rainfall	Female	Age 60 years and above	NR	NR
Sedyaningsih-Mamahit et al., 2002^a^	Indonesia	Water-borne	River water as primary source of cooking, bathing and human waste disposal	Female	Increasing age	NR	Rural
Wibawa et al., 2004	Indonesia	Water-borne, foodborne (zoonotic)	Unhygienic water, undercooked or uncooked pig meat and viscera	NR	50 − 59 years old	NR	NR
Surya et al., 2005	Indonesia	Foodborne (zoonotic)	Undercooked grilled pork	NA	NR	NR	NR
Wibawa et al., 2007	Indonesia	Zoonotic	Ingesting uncooked pig meat and viscera, and vegetable mixed with fresh blood from pigs	NR	NR	NR	NR
Utsumi et al., 2011	Indonesia	Zoonotic	Close contact with animals/or animal waste (pig), consuming uncooked or undercooked swine meat	NR	Age above 20 years	Swine farm workers	NR
Widasari et al., 2013	Indonesia	zoonotic	Close association with pigs (as domestic animals), consumption of raw pig viscera and fresh blood mixed with vegetables	NR	15 − 40 years old	Swine farm workers	NR
Holt et al., 2016	Lao PDR	Waterborne zoonotic	Unprotected water sources, Poor hygiene practice (open defecation practice, infrequent hand washing), high pig contact (pig slaughtering, handling offal/raw meat, drinking raw pig’s blood, pigs in household)	Male	children	NR	NR
Khounvisith et al., 2018	Lao PDR	Zoonotic	Feeding of pigs	Male	50 years and above	Pig farmers, Slaughterhouse workers	NR
Tritz et al., 2018	Lao PDR	zoonotic	Close contact with cattle, consumption of raw or undercooked meat, consumption of raw blood	Male	Increasing age	Livestock farmers	Rural
Seow et al., 1999	Malaysia	NR	NR	DNS	DNS	NR	Rural
Ng et al., 2000	Malaysia	Fecal-oral (waterborne)	NR	Female	DNS	NR	NR
Hudu et al., 2018	Malaysia	Zoonotic	NR	Male	61 years and above	NR	NR
Uchida et al., 1993**^a^**	Myanmar	Fecal-oral (waterborne)	NR	NR	NR	NR	NR
Wong et al., 2019	Singapore	Foodborne (zoonotic)	Consumption of pork and pig products	Male	55 years and above	NR	NR
Poovorawan et al., 1996	Thailand	NR	NR	Male	Increasing age	NR	NR
Hinjoy et al., 2013	Thailand	Foodborne (zoonotic), waterborne	Consumption of pig organs, household flood	Male	65 years and above	DNS	NR
Gonwong et al., 2014	Thailand	Foodborne (zoonotic),	Consumption of pork,	NA	-	NR	NR
Sa-nguanmoo et al., 2015	Thailand	Zoonotic	Contact with swine, pork consumption	DNS	21–50 years old	Swine farmers, animal transporters, abattoir workers, pork handlers	NR
Hau et al., 1999	Vietnam	Waterborne	DNS	DNS	50 years and above	NR	NR
Hoan et al., 2019	Vietnam	Zoonotic	Permanent contact with pig	NR	NR	Pork meat vendors, pig slaughterers, pig farmers	NR

a; outbreak investigation study, DNS; data not statistically significant, NR; not reported, PDR; peoples’ democratic republic.

### Circulating HEV genotypes in SEA

Data on prevalent HEV genotypes in SEA is summarized in Table 6. Studies on HEV genotypes are reported from six countries. These countries are; Cambodia, Indonesia, Malaysia, Singapore, Thailand, and Vietnam.

**Table 6.** HEV genotype distribution across Southeast Asia

country	Year of sampling	Sample	Amplified region	genotype	Isolate designation	GenBank accession number	PCR type used	Assay type used	Source
Cambodia	2010–2014	Serum sample of a 39 year old man	Full-length genome sequence	4	CVS-Sie10	LC042232	Nested RT-PCR	Takara Bio	Yamada et al., 2015
Cambodia	2010–2014	Serum sample of an adult woman	ORF1	3	-	-	Nested RT-PCR	-	Yamada et al., 2015
Cambodia	2014	Serum sample of a 28 year old man	ORF3	3	NA	LC102813	In-house qRT-PCR	QIAamp viral RNA mini kit	Nouhin et al., 2016
Indonesia	2003–2006	Serum sample of a 28 year old man with sporadic acute hepatitis E	ORF2 457 bp (location: 5,965–6,421)	4	BaliE03-46	NA	Nested RT-PCR	NA	Wibawa et al., 2007
Malaysia	2018	Serum samples from 8 patients with chronic hepatitis B, 5 males/6 females, 51->61 years old	ORF2 345 bp	4	HSA14, HSA23,HSA37, HSA45, HSA60, HSA69,HSA75 & HSA77	KX426575-KX426582	Nested RT-PCR	QIAamp viral RNA mini kit	Hudu et al., 2018
Singapore	2007–2016	143 serum samples,	ORF1 258 bp, ORF2 304 bp	1 (21 samples), 3 (121 samples) & 4 (1 sample)	-	-	Nested RT-PCR	Invitrogen	Wong et al., 2019
Thailand	2008–2009, 2011	5 serum samples of acute hepatitis E patients	ORF2 415 bp	3	TH-hu-SL5080, TH-hu-SL5683,TH-hu-SL66, TH-hu-SL94 & TH-hu-SL97	KF145136-KF145140	Semi nested RT-PCR	QIAamp viral RNA mini kit	Siripanyaphinyo et al., 2014
Vietnam	2012–2013	Serum sample of a HBV patient with LC and HCC	ORF1 306 bp, ORF2 497 bp	3	NA	NA	Nested RT-PCR	QIAamp viral RNA mini kit	Hoan et al., 2015

## Discussion

According to the United Nations (UN) geoscheme as well as the UN statistical division (UNSD) department, the subregion of South – eastern Asia is made up of 11 countries [47]. The list and characteristics of the countries that made up the SEA subregion are given in S3 File. There are two (Brunei and Singapore) developed (high – income) countries, and the remaining are developing (upper – middle, lower – middle and low – income) countries in the subregion. Of all the 35 articles included in the SR, there was no captured research in two (Brunei and Timor – Leste) out of the 11 countries. However, data was recorded in the remaining nine countries, indicating that HEV infection is prevalent in the region.

The endemicity of HEV infection in SEA is further confirmed by the seroprevalence data extracted from 32 publications with 33 studies in seven countries of the region (Table 2). Judging from the earliest recorded study, it is evident that HEV infection has been present in the subregion for at least two decades and a half [48]. HEV seroprevalence showed wide variations between countries, within the countries, and from one population to another. The highest recorded seroprevalence was 77.7% among the healthy population of lowland communities in Lao PDR [49]. On the other hand, the lowest rate was reported among urban blood donors in Malaysia, with 2% [50]. The wide variations seen in the HEV seroprevalence between countries in SEA is like the results obtained from Africa. In a similar SR conducted by Kim and colleagues in Africa, HEV seroprevalence varied by country from 0% (in Gabon) to 84.3% (in Egypt) [17]. Studies in Europe have also shown HEV seroprevalence variations between countries in that region [51,52]. However, the variations in Europe are not as wide as those seen in this SR study and those observed in Africa [51,52]. The reason for the seroprevalence variation by country could be due to several factors. These factors include, among others, the assay method employed, and publication year. The assay used for the seroprevalence study could explain the observed variation in seroprevalence across countries. These assays vary in their performances (sensitivity and specificity) thus may give results that differ from one study to another. There seems to be no consistency in the assay type used across the countries and even within countries. Different assays are employed within and across countries for determining the HEV seroprevalence. Several studies investigating performance of assays used in HEV seroprevalence studies have established that employed assay type is a predictor of seroprevalence [53–56]. Implying that type of assay used in a study can influence seroprevalence estimations. In one of the studies, a broad range (42%-96%) of sensitivity was reported for anti – HEV detection among the five assay types studied [53]. Another essential predictor for variation in HEV seroprevalence is the year of publication. As shown in the results of this SR, chronologic time could show either increasing or decreasing HEV prevalence. An example of declining HEV seroprevalence in a study investigating the chronological time was noted in one of the studies in Cambodia.

Where the comparison of IgG seroprevalence between 1996–2000 and 2016–2017 periods showed a significant decrease from 61.35 to 32.3%, respectively [57]. However, in Singapore, the situation was the reverse of what is observed in Cambodia. Here, IgG seroprevalence increased from 14% in 2007 to 35% in 2016 [58]. In addition to factors enumerated as influencing variation of seroprevalence between countries, cultural practices, eating habits, and whether a country is developed or developing [59] will affect seroprevalence within a particular country. The high rate HEV seroprevalence observed in this SR study indicates that HEV is hyperendemic in the subregion. The majority of countries in this study are developing with either low – income, lower – middle income, or upper – middle income economies. This could explain the high rate of HEV seroprevalence observed.

However, even in Singapore, a developed country with a high – income economy, the seroprevalence (of 10.5% – 22.3%) appeared relatively high [58,60]. This scenario goes to prove that the endemicity of HEV is no longer restricted to only developing countries, but even developed countries are no exception [51]. Other factors that may lead to HEV seroprevalence variation in the subregion include study type, sample size or frame [18], migration, tourism, and proportion of specific ethnic groups in a country [19]. Studies were conducted among susceptible and non – susceptible groups as well. Thus, results revealed that HEV seroprevalence also differs among different study populations. Results showed that seroprevalence tends to be higher among the susceptible groups across almost all the countries. In Cambodia, seroprevalence is between the range of 18% to 28.2% among the general population, healthy individuals and voluntary blood donors. Whereas, among the susceptible populations (patients with unexplained febrile illness) the seroprevalence is 30.1% [57,61–63]. The seroprevalence rate of the general population in Indonesia ranges from 5.9% to 18%, while the susceptible group (those living in previous outbreak areas) have a range of 18% to 59%. In Vietnam, the seroprevalence of the at risk group of individuals exposed to pigs is 53% and 31% among healthy populations. The results are similar in Lao PDR, Malaysia, and Singapore. This pattern of seroprevalence has also been reported in an SR of HEV epidemiology conducted in Africa [17].

Furthermore, evidence of HEV infection endemicity in the SEA is not limited to seroprevalence studies alone. Reports on sporadic cases also exist to buttress further the fact that hepatitis E is endemic in the subregion. Seven hundred and fifteen confirmed acute hepatitis E cases from 1996 to 2019 were reported in the subregion out of 7079 sampled patients. The seropositivity rate ranges from 1.1% [61] to 88% [64] amongst different age groups (Table 3). Acute hepatitis E cases in most countries are low despite the high rate of HEV seroprevalence in the respective countries. However, the clinical HEV infection of 88% in Indonesia is in tune with the high seroprevalence rate reported in the country [48,64].

Nonetheless, the low rate disparity indicates either a high rate of asymptomatic HEV infection cases or misdiagnoses/missed diagnoses in the subregion. The results obtained in this SR study is not dissimilar with what was observed elsewhere in an SR conducted in Africa and Europe [17,51]. These results are also in line with reports from several primary studies showing high asymptomatic HEV infection [65–67]. There are also suggestions that certain HEV genotypes may be responsible for more symptomatic (HEV – 1 and HEV – 2) and asymptomatic (HEV – 3) infections [68]. Therefore, a low rate of acute HEV infections within a particular region or country, if due to asymptomatic cases, may imply the prevalence of HEV genotype with a less virulent course. They are thereby resulting in more asymptomatic cases as opposed to presentation with symptoms.

Additionally, disease outbreaks are apparent indications of the occurrence of such disease in the location. Accordingly, HEV discovery was traced back to the historic 1955 epidemic of acute hepatitis in Delhi, India [69]. Ever since the first recorded outbreak, several HEV infection outbreaks have been reported in the developing countries of Africa and Asia. Likewise, in this SR, two outbreaks were identified in Myanmar [70] and Indonesia [64]. Table 4 showed that the Myanmar outbreak occurred in 1989, with 108 confirmed cases. Jaundice was the most frequent presenting symptom. However, the clinical attack rate and case fatality rate were not reported. The Indonesian epidemic occurred between 1997–1998, involving 110 confirmed cases with no mortality [64]. More females were affected than males, and the clinical attack rate was 19% [64]. Both Myanmar and Indonesia are developing nations with low income economies. Thus, reports of hepatitis E outbreak in these countries agree with established results of restriction of hepatitis E outbreaks to low income nations [69]. The trend of HEV outbreaks occurring in low income countries was also the same in Africa, as reported by Kim et al. (2015). Although, in Kim’s study, the frequency of the epidemics in Africa is more than what is reported in this study. In Kim’s study, more outbreaks were reported in almost alternate years. Whereas, in this study, only two outbreaks were identified and in two countries. The possible explanation for this could be that outbreaks in SEA are seldom reported in peer review journals.

Moreover, in addition to evaluating seroprevalence, clinical HEV infection, and outbreaks investigations, this study also looked at the associated risk factors, at risk groups, and the route of HEV transmission. It is believed that the evaluation of risk factors will provide a clue for the observed country- to -country, and regional differences in the seroprevalence. Thus, in addition to other enumerated factors such as the assay method, assessing the disease risk factors that prevail in each country or region will offer more clarity as to why the variations in seroprevalence. From the results of most studies included in this SR, there are several risk factors for HEV infection or seropositivity. These factors can be categorized based on risk practices, gender, age, occupation, and place of residence. Certain practices were identified as predisposing factors to HEV seroprevalence. The risk practices or conditions range from close contact with animals or animal waste, eating of uncooked or undercooked swine meet to blood transfusion (Table 5). In one of the studies, the eating habit of the population was attributed to the high anti – HEV antibody seropositivity. People in that community are known for consuming uncooked pig intestines and fresh blood mixed with vegetables [71]. Thus, dietary preference for eating raw or undercooked animal products, particularly pig liver, will predispose to a high rate of HEV seroprevalence in a region. Other recorded risk activities include poor hygienic practice, drinking fecal contaminated water, and human waste disposal into water bodies. The findings in this SR is in line with an SR conducted in some selected non – endemic countries. Wherein dietary preferences for uncooked liver and HEV contamination of food sources were identified as factors impacting HEV IgG seroprevalence [19]. The male gender and elderly age group were also identified as at – risk groups for high HEV seropositivity in most studies. However, some studies recorded no significant difference between male and female HEV seroprevalence.

Regarding sporadic HEV infection, male gender and advanced age were also reported to be more affected in most of the studies. Of the few studies that investigated the predisposition of places of residence to HEV seroprevalence all, identified rural residence as a risk factor. HEV seroprevalence was also noted to have a predilection to some individuals in certain occupations. Animal transporters, abattoir workers, slaughterhouse workers, swine farm workers, swine farmers, and livestock farmers were all acknowledged as at – risk occupations for HEV seropositivity. This study also identified several transmission routes for HEV infection from analyzed studies, as outlined in Table 5.

Consequently, a new categorization for the HEV transmission route based on the findings of this SR may not be out of place. So, transmission routes for HEV can be divided into three broad groups; non – zoonotic, zoonotic, and vertical transmission. The non – zoonotic transmission can be subdivided further into waterborne (due to fecal contamination), bloodborne, and person-to-person (direct contact) transmission. The zoonotic transmission can be either direct or indirect. Direct zoonotic transmission will entail transmission from animals to humans through; direct contact with animals, their fluids and secretions, or wastes. The indirect zoonotic transmission has three subdivisions. One, waterborne; from contamination of water with HEV infected animal waste. Two, foodborne; from consuming contaminated animal products of HEV infected animals. Three, bloodborne; the sources for bloodborne transmission for zoonotic and non – zoonotic could be any of these; – organ transplant, hemodialysis, blood transfusion, and intravenous drug administration/abuse. These bloodborne sources have already been established in several studies as avenues of HEV transmission [72,73].

For a complete outlook of HEV epidemiology in the subregion, this study also assessed the prevailing HEV genotypes. The most prevalent genotype in the subregion is HEV – 3, followed by HEV – 4 (Table 6). HEV – 3 being the most prevalent genotype, further justifies the observed inverse relationship between HEV seroprevalence and rate of clinical HEV infection detected in most countries. Also, interesting to note, is the prevalence of HEV – 1 in Singapore, a developed country. HEV – 1 is believed to be restricted to only developing countries, but here, in addition to HEV – 3 and HEV – 4, HEV – 1 was also reported in Singapore (Figure 4). However, the study alluded to the fact that HEV – 1 might be imported into the country by travelers or non – residents [58]. Another striking observation also is that despite recorded HEV outbreak in Indonesia, HEV – 1 and HEV – 2 were not reported in the country. Instead, HEV – 4 was the identified genotype and not the so – called epidemic genotypes (HEV – 1 and HEV – 2). Indonesia is a developing country with hygienic and sanitary challenges that favors HEV – 1 and HEV – 2 prevalence. However, other risk practices and conditions in favor of HEV – 4 and HEV – 3 also exist in the country. So, possibilities are that the epidemic genotypes are the most prevalent but yet to be identified. Another probability could be that HEV – 1 is most prevalent in the outbreak areas and HEV – 4 in non – epidemic areas. Also, there is the likelihood of a predominant genotype switch occurring in Indonesia from HEV – 1 to HEV – 4, since it is possible to have mixed genotype prevalence in a country. A similar situation also exists in China, another outbreak country. Previously, HEV – 1 was believed to be the most abundant genotype in China. However, most recent studies have reported HEV – 4 as the most prevalent [74].

This SR as well, identified knowledge gaps and limitations relating to HEV research and epidemiology. HEV study paucity was identified in some countries in the region. Of interest are the two countries (Brunei and Timor-Leste) that did not report any study. So, more studies are needed, particularly on sporadic infection and outbreak investigation in the subregion. Likewise, more seroprevalence studies on susceptible groups are required. Studies on susceptible groups such as pregnant women, immunocompromised, and hemodialysis patients are of a limited number in the subregion. To standardize HEV diagnostic method, more studies should be conducted examining the performance characteristics of different assay types. This type of investigation will allow for the adoption of assays with similar superior performance characteristics for future HEV diagnostic studies. Also, in conducting future investigations, global or regional standard generic protocols could be generated to be adopted for the research. This protocol will allow for harmonization and better comparison among different studies. A similar proposal for a standard protocol in HEV seroprevalence research has been made earlier [17]. The high rate of asymptomatic cases noted here may lead to underestimating the hepatitis E burden in the subregion. Thus, implementation of routine HEV screening in the hospitals, at least among the high risk groups, will be worthy.

Consequent to the above enumerations and findings, there is a need for recommendations. Recommendations that will help shape policy formulation toward effective control and prevention of hepatitis E in the subregion. A joint sub-regional/multilateral collaboration among member countries for the control of hepatitis E is needed. This collaboration will allow for harmonizing immigration and migration policies across the subregion. It will also work on the effective livestock movement and animal husbandry practices within the member nations. Within countries, there is a need for robust HEV control and prevention programmes. Starting from improved government intervention in the area of HEV research. Improved research will unravel the actual burden of hepatitis E. It may also lead to the development of novel vaccine(s) to prevent the disease. Also, governments at all levels should focus on improving decent basic hygiene. Concerted efforts should be made to provide safe drinking water. Ensure adherence to standard guidelines for public water supply. Also, the adoption of eco – friendly sanitation measures regarding sewage disposal (for both animal and human) in communities will help prevent the disease.

Furthermore, health education on improved personal hygiene and safe dietary preference behaviors will help HEV infection prevention and control. Discouraging the consumption of uncooked or undercooked animal products and contaminated beverages will have a great impact on HEV prevention. Enhancing surveillance for early outbreak detection and upgraded blood screening strategy will reduce epidemic impact and improve control.

### Strength and limitation of the study

This is the first SR study on the epidemiology of HEV infection in the SEA subregion. This SR is unique and robust in two aspects. One, the screening process was culminated with a vigorous critical appraisal to determine the included studies. This ensured that only quality studies are included for the SR and eliminated bias. Two, a comprehensive approach was adopted to include the most critical aspects of disease epidemiology as the study’s outcomes. However, the study is not without some limitations. There is the possibility of unintentional omission of relevant publications since only five databases were searched for this SR. Also, only publications in English language were considered eligible for inclusion in the study.

## Conclusion

Hepatitis E is highly endemic in SEA, as evidenced by the high rate of HEV seroprevalence and recorded sporadic HEV infections across the countries. Even though there are possibilities of underestimation of the disease due to the high rate of asymptomatic infections. Therefore, there is a need for determined efforts toward determining the actual disease burden for effective prevention and control.

## Supplementary Material

Supplemental Material
