# Supplementary material for: A systematic review of the epidemiology of Hepatitis E virus infection in South – Eastern Asia
Source: Virulence. 2020 Dec 29;12(1):114–29. doi: 10.1080/21505594.2020.1865716 (PMC7781573; doi:10.1080/21505594.2020.1865716)
Supplement: Supplemental Material [file KVIR_A_1865716_SM5220.zip › supplement/S1_File.docx]

S1 File. Study protocol

**Tittle: Protocol for a systematic review of the epidemiology of hepatitis E virus Infection in Southeast Asia**

**Registration:** Not registered

Authors: Yakubu Egigogo Raji ^1,3^, Ooi Peck Toung ^2^ Niazlin Mohd Taib ^1^ Ooo Onna and Zamberi Bin Sekawi ^1,^*

**^1^** Department of Medical Microbiology and Parasitology, Faculty of Medicine and Health Sciences, Universiti Putra Malaysia 1; [yakuburajiegigogo@gmail.com](mailto:yakuburajiegigogo@gmail.com) (Y.R); [niazlin@upm.edu.my](mailto:niazlin@upm.edu.my) (N.T); [zamberi@upm.edu.my](mailto:zamberi@upm.edu.my) (Z.S)

**^2^** Department of Veterinary Clinical Studies Faculty of Veterinary Medicine, Universiti Putra Malaysia 2; [ooi@upm.edu.my](mailto:ooi@upm.edu.my) (O.T)

**^3^** Department of Pathology, Clinical Microbiology Unit College of Health Sciences Ibrahim Badamasi Babangida University Lapai Nigeria; [binrajikawu@yahoo.co.uk](mailto:binrajikawu@yahoo.co.uk) (Y.R)

***** Correspondence: [zamberi@upm.edu.my](mailto:zamberi@upm.edu.my) ; Tel.: +600389410130

**Authors’ contributions:** Conception of research idea (ZS), Literature review (YR), Research protocol design (YR), Study appraisal (ZS, OT and NT), Data extraction (YR, NT and ZS), Data analysis and interpretation of results (ZS, OT, NT and YR), Manuscript drafting (YR), and review of initial and final draft of manuscript (ZS, OT and NT)

**Source of Support:** Universiti Putra Malaysia

**Sponsor:** Universiti Putra Malaysia

**Role of sponsor:** The sponsor did not play any role in the development of the study protocol.

**Introduction**

Hepatitis E is an emerging viral disease with an increasing international public health challenge. The disease is globally distributed but endemic in most part of Asia and Africa. The global disease burden is estimated to be 2.3 billion and the leading cause of acute viral hepatitis particularly in the developing nations ([Teshale and Hu, 2011](#_ENREF_12), [Melgaço et al., 2018](#_ENREF_7)). Annually, hepatitis E virus is responsible for over 20 million new cases, 3 million symptomatic hepatitis and about 40,000 to 160,000 deaths ([Pischke et al., 2017](#_ENREF_9), [Webb and Dalton, 2019](#_ENREF_13), [Gupta and Agarwala, 2018](#_ENREF_4)). Hepatitis E virus has been a major cause of epidemic hepatitis in the lower income countries of Asia and Africa ([Kmush et al., 2013](#_ENREF_6)). Though, the number of HEV cases have been increasing even in the developed countries in recent years ([Clemente-Casares et al., 2016](#_ENREF_1)). Before now it was believed that the disease is transmitted through faecal – oral route only thus, explaining why the disease is endemic in developing regions with poor sanitary conditions ([Rein et al., 2012](#_ENREF_10), [Sreenivasan, 1978](#_ENREF_11), [De Cock et al., 1987](#_ENREF_2)). Additionally, hepatitis E was previously thought to be a self-limiting infectious disease that does not lead to severe or chronic disease ([Emerson and Purcell, 2003](#_ENREF_3)). However, an increase in HEV research in recent time has resulted in a pattern shift in the understanding of HEV epidemiology. Hepatitis E is now believed to have evolved from an enteric self – limiting illness to a multifactorial, multi – risk and chronic disease. It is believed to have changed from a regional disease to a global disease. Despite these growing concerns about this emerging disease with its epidemiologic and clinical consequences, there is a strong indication that the disease is still underestimated. The disease is either missed or misdiagnosed by clinicians in the hospitals. Routine screenings are rarely done for HEV in the hospitals. Not even for the high-risk groups such as the pregnant women and the immunocompromised.

Therefore, evaluation of the disease epidemiology in South East Asian countries through a systematic review will assist towards unravelling the burden and the epidemiological trend of this emerging infectious disease that has a growing global risk specifically in the face of increase global travels, livestock trade and conflict. Thus, providing information that will form the bases for implementing evidence – based policies aimed at reducing the burden of the disease in the region.

**Objectives:**

Aim: is to evaluate the epidemiology of the HEV infection in south east Asia by reviewing and summarising pertinent peer – reviewed literatures.

Specific objectives include:

1. To explore the rate of HEV infection (seroprevalence, outbreaks, sporadic cases) by combining the results of numerous relevant similar observational studies in south east Asian countries.

2. To assess the mode of disease transmission, risk factors, at risk groups and identify circulating HEV genotypes for a better understanding of the epidemiology of the infection in south east Asia.

3. To identify knowledge gaps in HEV studies and make recommendations for the purpose of implementing measures to prevent HEV infection in at – risk population particularly and the general population in the south east Asia region.

**Methods**

- Inclusion criteria
  - - Study location; studies conducted in any of the South East Asian countries. South East Asia countries as used by the United Nations (United Nations Statistics Division).
    - Context; the study will include all observational studies (cross – sectional studies, intervention studies, cohort studies, case – control studies, case series, longitudinal prevalence studies, seroprevalence studies, prevalence surveys) that report the prevalence (seroprevalence) of HEV infection (IgG, IgM) in any of the study locations.
    - Time period; no limitations placed on the year of publication.
    - Language of publication; English.
    - Participants; Susceptible groups (pregnant women, patients with co – infection, patients with chronic disease, animal farm owners, farm workers, veterinary officers, displaced persons, prisoners, homeless, sex workers, illicit drug users, rural dwellers) and non – susceptible groups (healthy general population, healthy blood donors, urban residents).
    - Age; any age range.
- Exclusion criteria
  - - Studies that did not clearly separate the prevalence of HEV infection from other viral diseases and studies conducted in animals only.
    - Studies conducted in non – south east Asian population.
    - Studies with suboptimal methodology and studies covering topics other than HEV epidemiology (laboratory studies on pathogenesis of diseases, molecular biology).
    - Studies without clear description of population, serological assay, time period and place of sample collection.
    - Case reports, letters, books, dissertations, review articles, unpublished reports and conference papers.

**Search strategy:**

The search strategy will embrace the assessment of all relevant literature citations captured through the application of the search algorithm in five electronic bibliographic databases. Also, a grey literature search will be conducted via hand searching references of selected (review) articles and conference proceedings. Additionally, a corresponding internet search will be done in Google scholar, Google and Yahoo applying the same algorithm used in the bibliographic database search.

**Databases**

The five Selected databases to be used include Scopus, Science Direct, PubMed, MEDLINE and ASEAN Citation Index. The electronic search strategy will be as follows:

**Library/Database:** Scopus

**Date of Search:** March 22, 2020

**Search String:**  Advanced search ((“Hepatitis E Virus” OR “HEV infection” OR “HEV” OR “Viral Hepatitis E” OR “Hepatitis E” OR “Hepatitis E virus infection” OR “Hepatitis E antibodies”) AND (Seroepidem* OR “Prevalence” OR Epidem* OR “Survey” OR “Seroprevalence”) (“Indonesia” OR “Vietnam” OR “Thailand” OR “Singapore” OR “Malaysia” OR “Philippines” OR “Cambodia” OR “Myanmar” OR “Burma” OR “Laos” OR “Brunei” OR “Timor-Leste”))

**# Hits:2,438**

**Library/Database:** Science Direct

**Date of Search:** March 22, 2020

**Search String:**  Articles (“Hepatitis E Virus” OR “HEV infection” OR “HEV” OR “Viral Hepatitis E” OR “Hepatitis E” OR “Hepatitis E virus infection” OR “Hepatitis E antibodies”) AND (Seroepidem* OR “Prevalence” OR Epidem* OR “Survey” OR “Seroprevalence”)

Title, Abstract, or Author – specified Keywords (“Indonesia” OR “Vietnam” OR “Thailand” OR “Singapore” OR “Malaysia” OR “Philippines” OR “Cambodia” OR “Myanmar” OR “Burma” OR “Laos” OR “Brunei” OR “Timor-Leste”)

**# Hits: 51**

**Library/Database:** MEDLINE

**Date of Search:** March 22, 2020

**Search String:** All fields ((“Hepatitis E Virus” OR “HEV infection” OR “HEV” OR “Viral Hepatitis E” OR “Hepatitis E” OR “Hepatitis E virus infection” OR “Hepatitis E antibodies”) AND (Seroepidem* OR “Prevalence” OR Epidem* OR “Survey” OR “Seroprevalence”) AND (“Indonesia” OR “Vietnam” OR “Thailand” OR “Singapore” OR “Malaysia” OR “Philippines” OR “Cambodia” OR “Myanmar” OR “Burma” OR “Laos” OR “Brunei” OR “Timor-Leste”))

**# Hits: 1,478**

**Database:** PubMed

**Date of Search:** March 22, 2020

**Search String: (**(“Hepatitis E Virus” OR “HEV infection” OR “HEV” OR “Viral Hepatitis E” OR “Hepatitis E” OR “Hepatitis E virus infection” OR “Hepatitis E antibodies”) AND (Seroepidem* OR “Prevalence” OR Epidem* OR “Survey” OR “Seroprevalence”) AND (“Indonesia” OR “Vietnam” OR “Thailand” OR “Singapore” OR “Malaysia” OR “Philippines” OR “Cambodia” OR “Myanmar” OR “Burma” OR “Laos” OR “Brunei” OR “Timor-Leste”))

**# Hits: 184**

**Database:** ASEAN Citation Index

**Date of Search:** March 22, 2020

**Search String:** All fields ((“Hepatitis E Virus” OR “HEV infection” OR “HEV” OR “Viral Hepatitis E” OR “Hepatitis E” OR “Hepatitis E virus infection” OR “Hepatitis E antibodies”) AND (Seroepidem* OR “Prevalence” OR Epidem* OR “Survey” OR “Seroprevalence”) AND (“Indonesia” OR “Vietnam” OR “Thailand” OR “Singapore” OR “Malaysia” OR “Philippines” OR “Cambodia” OR “Myanmar” OR “Burma” OR “Laos” OR “Brunei” OR “Timor-Leste”))

**# Hits: 0**

**# Hits:**

Total # of citations prior to de-duplication: 4,151

Total # of citations after 1^st^ round of de-duplication in Excel: 3,368

Total # citations added from manual search: 6

**Data management**

The search strategy will be compiled and de-duplicated in an MS Excel spreadsheet. All steps of the systematic review from screening to data extraction will be carried out on Excel spreadsheet. The final dataset on the MS Excel spreadsheet will then be subjected to further analysis.

**Selection process**

The study selection will be conducted by two independent reviewers and a third reviewer will decide about uncertainties.

**Data collection process**

Extraction of data will be conducted simultaneously with the full text searching. The relevant information will be extracted from each article included and recorded immediately in the data extraction file (MS Excel). This will be carried out by two independent reviewers and two others will check the information.

**Outcomes:**

Patients with serological evidence of HEV exposure IgG or IgM (from seroprevalence, outbreaks, sporadic cases studies).

**Other outcomes**

Identify mode of HEV transmission, risk factors, at risk groups and circulating genotypes.

**Risk of Bias (quality) assessment**

Each article to be included for analysis will be a relevant article that meets the inclusion and exclusion criteria. The quality of each article will be evaluated based on the prevalence critical appraisal instrument developed by ([Munn et al., 2014](#_ENREF_8)) for seroprevalence studies and the critical appraisal check list by ([Joanna Briggs Institute, 2017](#_ENREF_5)) for case series and outbreak studies. The 10 questions used in each of the critical appraisal instrument can be answered either with Yes, No, Unclear, or not applicable. Articles with ≤60% score, or ≥3 U, will be considered to have failed the quality assessment test and will not be included in the study.

**References**

CLEMENTE-CASARES, P., RAMOS-ROMERO, C., RAMIREZ-GONZALEZ, E. & MAS, A. 2016. Hepatitis E virus in industrialized countries: the silent threat. *BioMed research international,* 2016.

DE COCK, K. M., BRADLEY, D. W., SANDFORD, N. L., GOVINDARAJAN, S., MAYNARD, J. E. & REDEKER, A. G. 1987. Epidemic non-A, non-B hepatitis in patients from Pakistan. *Annals of Internal Medicine,* 106**,** 227-230.

EMERSON, S. U. & PURCELL, R. H. 2003. Hepatitis E virus. *Reviews in medical virology,* 13**,** 145-154.

GUPTA, E. & AGARWALA, P. 2018. Hepatitis E virus infection: An old virus with a new story! *Indian journal of medical microbiology,* 36**,** 317.

JOANNA BRIGGS INSTITUTE 2017. The Joanna Briggs Institute Critical Appraisal tools for use in JBI systematic reviews–checklist for case series. 2016.

KMUSH, B., WIERZBA, T., KRAIN, L., NELSON, K. & LABRIQUE, A. B. 2013. Epidemiology of hepatitis E in low- and middle-income countries of Asia and Africa. *Semin Liver Dis,* 33**,** 15-29.

MELGAÇO, J. G., GARDINALI, N. R., DE MELLO, V. D. M., LEAL, M., LEWIS-XIMENEZ, L. L. & PINTO, M. A. 2018. Hepatitis E: Update on Prevention and Control. *BioMed research international,* 2018**,** 5769201-5769201.

MUNN, Z., MOOLA, S., RIITANO, D. & LISY, K. 2014. The development of a critical appraisal tool for use in systematic reviews addressing questions of prevalence. *International journal of health policy and management,* 3**,** 123-128.

PISCHKE, S., HARTL, J., PAS, S. D., LOHSE, A. W., JACOBS, B. C. & VAN DER EIJK, A. A. 2017. Hepatitis E virus: Infection beyond the liver? *Journal of hepatology,* 66**,** 1082-1095.

REIN, D. B., STEVENS, G. A., THEAKER, J., WITTENBORN, J. S. & WIERSMA, S. T. 2012. The global burden of hepatitis E virus genotypes 1 and 2 in 2005. *Hepatology,* 55**,** 988-997.

SREENIVASAN, M. 1978. Epidemiological investigations of an outbreak of infectious hepatitis in Ahmedabad city during 1975-76. *Indian Journal of Medical Research,* 67**,** 197-206.

TESHALE, E. H. & HU, D. J. 2011. Hepatitis E: Epidemiology and prevention. *World journal of hepatology,* 3**,** 285-291.

WEBB, G. W. & DALTON, H. R. 2019. Hepatitis E: an underestimated emerging threat. *Therapeutic advances in infectious disease,* 6**,** 2049936119837162-2049936119837162.
