# Supplementary material for: A systematic review of the epidemiology of Hepatitis E virus infection in South – Eastern Asia
Source: Virulence. 2020 Dec 29;12(1):114–29. doi: 10.1080/21505594.2020.1865716 (PMC7781573; doi:10.1080/21505594.2020.1865716)
Supplement: Supplemental Material [file KVIR_A_1865716_SM5220.zip › supplement/S1_Table.docx]

S1 Tile: Quality Assessment of Studies that meet the Inclusion and Exclusion Criteria of the SR

Table 1: All Seroprevalence studies (using the Prevalence Critical Appraisal Instrument by the JBI) ([Munn et al., 2014](#_ENREF_2)).

Note: Articles with ≤60% score, or ≥3 U, will not be included in the study.

| S/N | Study | Q1 | Q2 | Q3 | Q4 | Q5 | Q6 | Q7 | Q8 | Q9 | Q10 | Total |  |  |  |
| --- | --- | --- | --- | --- | --- | --- | --- | --- | --- | --- | --- | --- | --- | --- | --- |
|  |  |  |  |  |  |  |  |  |  |  |  | Y | N | U | NA |
| 1 | Hinjoy et al., 2013 | Y | Y | Y | Y | Y | Y | Y | Y | Y | Y | 10 | 0 | 0 | 0 |
| 2 | Sa-nguanmoo et al., 2015 | Y | Y | N | Y | Y | Y | Y | U | Y | Y | 8 | 1 | 1 | 0 |
| 3 | Jutavijittum et al., 2000 | Y | U | U | Y | Y | Y | Y | U | Y | U | 6 | 0 | 4 | 0 |
| 4 | Poovorawan et al., 1996 | Y | Y | U | Y | Y | Y | Y | N | N | Y | 7 | 2 | 1 | 0 |
| 5 | Myint et al., 2007 | Y | Y | U | Y | Y | Y | Y | Y | U | U | 7 | 0 | 3 | 0 |
| 6 | Pilakasiri et al., 2009 | Y | Y | Y | Y | Y | Y | Y | Y | Y | Y | 10 | 0 | 0 | 0 |
| 7 | Jupattanasin et al., 2019 | Y | Y | Y | Y | Y | Y | Y | Y | Y | Y | 10 | 0 | 0 | 0 |
| 8 | Khounvisith et al., 2018 | Y | Y | Y | Y | Y | Y | Y | Y | Y | Y | 10 | 0 | 0 | 0 |
| 9 | Tritz et al., 2018 | Y | Y | Y | Y | Y | Y | Y | Y | Y | Y | 10 | 0 | 0 | 0 |
| 10 | Lorenzo et al., 2015 | Y | Y | Y | N | Y | Y | Y | U | N | N | 6 | 3 | 1 | 0 |
| 11 | Chow et al., 1996 | Y | Y | U | Y | Y | Y | Y | U | N | Y | 8 | 1 | 1 | 0 |
| 12 | Wong et al., 2019 | Y | Y | U | N | Y | Y | Y | Y | Y | NA | 7 | 1 | 1 | 1 |
| 13 | Tran et al., 2003 | Y | Y | U | Y | N | Y | Y | Y | N | Y | 7 | 2 | 1 | 0 |
| 14 | Hau et al., 1999 | Y | Y | Y | Y | Y | Y | Y | Y | Y | Y | 10 | 0 | 0 | 0 |
| 15 | Hoan et al., 2019 | Y | Y | Y | Y | Y | Y | Y | Y | Y | Y | 10 | 0 | 0 | 0 |
| 16 | Hoan et al., 2015 | Y | Y | Y | Y | Y | Y | Y | Y | Y | Y | 10 | 0 | 0 | 0 |
| 17 | Berto et al., 2018 | Y | Y | Y | Y | Y | Y | Y | Y | Y | Y | 10 | 0 | 0 | 0 |
| 18 | Nouhin et al., 2015 | Y | Y | Y | Y | Y | Y | Y | Y | Y | Y | 10 | 0 | 0 | 0 |
| 19 | Yamada et al., 2015 | Y | Y | Y | Y | Y | Y | Y | Y | Y | Y | 10 | 0 | 0 | 0 |
| 20 | Nouhin et al., 2016 | Y | Y | U | Y | Y | y | Y | Y | U | U | 8 | 0 | 2 | 0 |
| 21 | Nouhin et al., 2019 | Y | NA | Y | Y | Y | Y | Y | Y | Y | NA | 8 | 0 | 0 | 2 |
| 22 | Ng et al., 2000 | Y | U | Y | Y | Y | Y | Y | Y | Y | Y | 9 | 0 | 1 | 0 |
| 23 | Seow et al., 1999 | Y | Y | U | Y | Y | Y | Y | U | Y | Y | 8 | 0 | 2 | 0 |
| 24 | Hudu et al., 2018 | Y | Y | U | Y | Y | Y | Y | Y | Y | Y | 9 | 0 | 1 | 0 |
| 25 | Wong et al., 2020 | Y | Y | U | Y | Y | Y | Y | N | U | U | 6 | 1 | 3 | 0 |
| 26 | Surya et al., 2005 | Y | Y | U | Y | Y | Y | Y | Y | Y | Y | 9 | 0 | 1 | 0 |
| 27 | Utsumi et al., 2011 | Y | Y | U | Y | Y | Y | Y | Y | Y | Y | 9 | 0 | 1 | 0 |
| 28 | Wibawa et al., 2004 | Y | Y | Y | Y | Y | Y | Y | Y | Y | Y | 10 | 0 | 0 | 0 |
| 29 | Widasari et al., 2013 | Y | Y | U | Y | Y | Y | Y | Y | Y | Y | 9 | 0 | 1 | 0 |
| 30 | Achwan et al., 2007 | Y | Y | U | Y | Y | Y | Y | N | Y | Y | 8 | 1 | 1 | 0 |
| 31 | Louisirirotchanakul et al., 2002 | Y | Y | U | Y | Y | U | U | U | Y | Y | 6 | 0 | 4 | 0 |
| 32 | Gonwong et al., 2014 | Y | Y | Y | Y | Y | Y | Y | Y | Y | Y | 10 | 0 | 0 | 0 |
| 33 | Holt et al., 2016 | Y | Y | Y | Y | Y | Y | Y | Y | Y | Y | 10 | 0 | 0 | 0 |
| 34 | Corwin et al., 1995 | Y | Y | Y | Y | Y | Y | Y | N | Y | Y | 9 | 1 | 0 | 0 |

Q1-10: Question1 – 10; Y: Yes; N: No; U: Unclear; NA: Not Applicable

Table 2: All Case series and Epidemic investigation studies (using the check list for case series by the JBI; a critical appraisal tool) ([Joanna Briggs Institute, 2017](#_ENREF_1)).

Note: Articles with ≤60% score, or ≥3 U, will not be included in the study.

| S/N | Study | Q1 | Q2 | Q3 | Q4 | Q5 | Q6 | Q7 | Q8 | Q9 | Q10 | Total |  |  |  |
| --- | --- | --- | --- | --- | --- | --- | --- | --- | --- | --- | --- | --- | --- | --- | --- |
|  |  |  |  |  |  |  |  |  |  |  |  | Y | N | U | NA |
| 1 | Siripanyaphinyo et al., 2014 | Y | Y | U | Y | Y | Y | Y | N | Y | Y | 8 | 1 | 1 | 0 |
| 2 | Uchida et al., 1993 | Y | Y | Y | Y | Y | Y | Y | N | Y | NA | 8 | 1 | 0 | 0 |
| 3 | Corwin et al., 1996 | Y | Y | Y | Y | Y | Y | N | N | Y | Y | 8 | 2 | 0 | 0 |
| 4 | Sedyaningsih-Mamahit et al., 2002 | Y | Y | Y | Y | Y | Y | N | NA | Y | Y | 8 | 1 | 0 | 1 |
| 5 | Wibawa et al., 2007 | Y | Y | Y | Y | Y | Y | N | N | Y | Y | 8 | 2 | 0 | 0 |
| 6 | Gloriani-Barzaga et al., 1997 | Y | Y | Y | Y | Y | Y | Y | Y | Y | U | 9 | 0 | 1 | 0 |
| 7 | Chow et al., 1997 | U | Y | Y | Y | Y | U | U | Y | Y | U | 6 | 0 | 4 | 0 |

**References**

JOANNA BRIGGS INSTITUTE 2017. The Joanna Briggs Institute Critical Appraisal tools for use in JBI systematic reviews–checklist for case series. 2016.

MUNN, Z., MOOLA, S., RIITANO, D. & LISY, K. 2014. The development of a critical appraisal tool for use in systematic reviews addressing questions of prevalence. *International journal of health policy and management,* 3**,** 123-128.
