# Supplementary material for: A systematic review of the epidemiology of Hepatitis E virus infection in South – Eastern Asia
Source: Virulence. 2020 Dec 29;12(1):114–29. doi: 10.1080/21505594.2020.1865716 (PMC7781573; doi:10.1080/21505594.2020.1865716)
Supplement: Supplemental Material [file KVIR_A_1865716_SM5220.zip › supplement/S2_File.docx]

**S2 File: PRISMA checklist**

| Item | Ref. page |
| --- | --- |
| Identify the report as a systematic review, meta-analysis, or both. | 1 |
| Provide a structured summary including, as applicable: background; objectives; data sources; study eligibility  criteria, participants, and interventions; study appraisal and synthesis methods; results; limitations; conclusions  and implications of key findings; systematic review registration number. | 2 |
| Describe the rationale for the review in the context of what is already known. | 3-4 |
| Provide an explicit statement of questions being addressed with reference to participants, interventions,  comparisons, outcomes, and study design (PICOS). | 5-6 |
| Indicate if a review protocol exists, if and where it can be accessed (e.g., Web address), and, if available, provide  registration information including registration number. | 5 |
| Specify study characteristics (e.g., PICOS, length of follow-up) and report characteristics (e.g., years considered,  language, publication status) used as criteria for eligibility, giving rationale. | 5-7 |
| Describe all information sources (e.g., databases with dates of coverage, contact with study authors to identify  additional studies) in the search and date last searched. | 7 |
| Present full electronic search strategy for at least one database, including any limits used, such that it could be  repeated. | 7 |
| State the process for selecting studies (i.e., screening, eligibility, included in systematic review, and, if applicable,  included in the meta-analysis). | 5-7 |
| Describe method of data extraction from reports (e.g., piloted forms, independently, in duplicate) and any  processes for obtaining and confirming data from investigators. | 8 |
| List and define all variables for which data were sought (e.g., PICOS, funding sources) and any assumptions and  simplifications made. | 9-11 |
| Describe methods used for assessing risk of bias of individual studies (including specification of whether this was  done at the study or outcome level), and how this information is to be used in any data synthesis. | 8 |
| State the principal summary measures (e.g., risk ratio, difference in means). | 10-11 |
| Describe the methods of handling data and combining results of studies, if done, including measures of  consistency (e.g., I2) for each meta-analysis. | 11 |
| Specify any assessment of risk of bias that may affect the cumulative evidence (e.g., publication bias, selective  reporting within studies). | 9 |
| Describe methods of additional analyses (e.g., sensitivity or subgroup analyses, meta-regression), if done,  indicating which were pre-specified | 10-11 |
| Give numbers of studies screened, assessed for eligibility, and included in the review, with reasons for exclusions  at each stage, ideally with a flow diagram. | 12 |
| For each study, present characteristics for which data were extracted (e.g., study size, PICOS, follow-up period)  and provide the citations. | S4 |
| Present data on risk of bias of each study and, if available, any outcome-level assessment (see Item 12). | S8 |
| For all outcomes considered (benefits or harms), present, for each study: (a) simple summary data for each  intervention group and (b) effect estimates and confidence intervals, ideally with a forest plot | S6 |
| Present results of each meta-analysis done, including confidence intervals and measures of consistency | 19-21 |
| Present results of any assessment of risk of bias across studies (see Item 15). | 25 |
| Give results of additional analyses, if done (e.g., sensitivity or subgroup analyses, meta-regression [see Item 16]). | 22-23 |
| Summarize the main findings including the strength of evidence for each main outcome; consider their relevance to key groups (e.g., health care providers, users, and policy makers). | 26-28 |
| Discuss limitations at study and outcome level (e.g., risk of bias), and at review level (e.g., incomplete retrieval of  identified research, reporting bias). | 29-31 |
| Provide a general interpretation of the results in the context of other evidence, and implications for future  research | 31-32 |
| Describe sources of funding for the systematic review and other support (e.g., supply of data); role of funders for  the systematic review. | 33 |
