# Supplementary material for: A systematic review of the epidemiology of Hepatitis E virus infection in South – Eastern Asia
Source: Virulence. 2020 Dec 29;12(1):114–29. doi: 10.1080/21505594.2020.1865716 (PMC7781573; doi:10.1080/21505594.2020.1865716)
Supplement: Supplemental Material [file KVIR_A_1865716_SM5220.zip › supplement/S3_File.docx]

**S3 File: List and Characteristics of South-east Asian Countries (According to United Nations Statistics Division)**

| **S/N** | **Countries** | **LDC** | **LLDC** | **SIDS** | **World Bank Income Group** |
| --- | --- | --- | --- | --- | --- |
| 1 | Brunei Darussalam |  |  |  | High |
| 2 | Cambodia | X |  |  | Low |
| 3 | Indonesia |  |  |  | Low |
| 4 | Lao People’s Democratic Republic | X | X |  | Low |
| 5 | Malaysia |  |  |  | Upper Middle |
| 6 | Myanmar | X |  |  | Low |
| 7 | Philippines |  |  |  | Lower Middle |
| 8 | Singapore |  |  | X | High |
| 9 | Thailand |  |  |  | Lower Middle |
| 10 | Timor-Leste |  |  |  | Low |
| 11 | Vietnam |  |  |  | Low |

LDC: Least developed countries; LLDC: Landlocked developing countries; SIDS: Small island developing States

United Nations, S.D. *Millennium Development Indicators: World and regional groupings*. 2003 [cited 2020 February, 6]; Available from: https://unstats.un.org/unsd/mi/asia.htm.
