# Supplementary material for: A systematic review of the epidemiology of Hepatitis E virus infection in South – Eastern Asia
Source: Virulence. 2020 Dec 29;12(1):114–29. doi: 10.1080/21505594.2020.1865716 (PMC7781573; doi:10.1080/21505594.2020.1865716)
Supplement: Supplemental Material [file KVIR_A_1865716_SM5220.zip › supplement/S4_File.docx]

S4 File: List of studies that meet Inclusion and exclusion criteria.

1. ACHWAN, W. A., MUTTAQIN, Z., ZAKARIA, E., DEPAMEDE, S. A., SUMOHARJO, S., TSUDA, F., TAKAHASHI, K., ABE, N. & MISHIRO, S. 2007. Epidemiology of hepatitis B, C, and E viruses and human immunodeficiency virus infections in Tahuna, Sangihe-Talaud Archipelago, Indonesia. *Intervirology,* 50**,** 408-411.

2. BERTO, A., PHAM, H., THAO, T., VY, N., CADDY, S., HIRAIDE, R., TUE, N., GOODFELLOW, I., CARRIQUE‐MAS, J. & THWAITES, G. 2018. Hepatitis E in southern Vietnam: Seroepidemiology in humans and molecular epidemiology in pigs. *Zoonoses and public health,* 65**,** 43-50.

3. CHOW, W., LEE, A., LIM, G., CHEONG, W., CHONG, R., TAN, C., YAP, C., OON, C. & NG, H. 1997. Acute viral hepatitis E: clinical and serologic studies in Singapore. *Journal of clinical gastroenterology,* 24**,** 235-238.

4. CHOW, W., NG, H., LIM, G. & OON, C. 1996. Hepatitis E in Singapore-a seroprevalence study. *Singapore medical journal,* 37**,** 579-582.

5. CORWIN, A., DAI, T., DUC, D. D., SUU, P., VAN, N. T., HA, L. D., JANICK, M., KANTI, L., SIE, A. & SODERQUIST, R. 1996. Acute viral hepatitis in Hanoi, Viet Nam. *Transactions of the Royal Society of Tropical Medicine and Hygiene,* 90**,** 647-648.

6. CORWIN, A., JAROT, K., LUBIS, I., NASUTION, K., SUPARMAWO, S., SUMARDIATI, A., WIDODO, S., NAZIR, S., ORNDORFF, G. & CHOI, Y. 1995. Two years' investigation of epidemic hepatitis E virus transmission in West Kalimantan (Borneo), Indonesia. *Transactions of the Royal Society of Tropical Medicine and Hygiene,* 89**,** 262-265.

7. GLORIANI-BARZAGA, N., CABANBAN, A., GRAHAM, R. R. & FLORESE, R. H. 1997. Hepatitis E virus infection diagnosed by serology: a report of cases at the San Lazaro Hospital, Manila. *Phil J Microbiol Infect Dis,* 26**,** 169-172.

8. GONWONG, S., CHUENCHITRA, T., KHANTAPURA, P., ISLAM, D., SIRISOPANA, N. & MASON, C. J. 2014. Pork consumption and seroprevalence of hepatitis E virus,Thailand, 2007-2008. *Emerging infectious diseases,* 20**,** 1531-1534.

9. HAU, C. H., HIEN, T. T., TIEN, N., KHIEM, H. B., SAC, P. K., NHUNG, V. T., LARASATI, R. P., LARAS, K., PUTRI, M. P. & DOSS, R. 1999. Prevalence of enteric hepatitis A and E viruses in the Mekong River delta region of Vietnam. *The American journal of tropical medicine and hygiene,* 60**,** 277-280.

10. HINJOY, S., NELSON, K. E., GIBBONS, R., JARMAN, R., MONGKOLSIRICHAIKUL, D., SMITHSUWAN, P., FERNANDEZ, S., LABRIQUE, A. B. & PATCHANEE, P. 2013. A cross‐sectional study of hepatitis E virus infection in healthy people directly exposed and unexposed to pigs in a rural community in northern Thailand. *Zoonoses and public health,* 60**,** 555-562.

11. HOAN, N. X., HUY, P. X., SY, B. T., MEYER, C. G., SON, T. V., BINH, M. T., GIANG, D. P., TU ANH, D., BOCK, C.-T. & WANG, B. High Hepatitis E virus (HEV) Positivity Among Domestic Pigs and Risk of HEV Infection of Individuals Occupationally Exposed to Pigs and Pork Meat in Hanoi, Vietnam. Open forum infectious diseases, 2019. Oxford University Press US, ofz306.

12. HOAN, N. X., VAN TONG, H., HECHT, N., SY, B. T., MARCINEK, P., MEYER, C. G., TOAN, N. L., KURRECK, J., KREMSNER, P. G. & BOCK, C.-T. 2015. Hepatitis E virus superinfection and clinical progression in hepatitis B patients. *EBioMedicine,* 2**,** 2080-2086.

13. HOLT, H. R., INTHAVONG, P., KHAMLOME, B., BLASZAK, K., KEOKAMPHE, C., SOMOULAY, V., PHONGMANY, A., DURR, P. A., GRAHAM, K. & ALLEN, J. 2016. Endemicity of zoonotic diseases in pigs and humans in lowland and upland Lao PDR: identification of socio-cultural risk factors. *PLoS neglected tropical diseases,* 10**,** e0003913.

14. HUDU, S. A., NIAZLIN, M. T., NORDIN, S. A., HARMAL, N. S., TAN, S. S., OMAR, H., SHAHAR, H., MUTALIB, N. A. & SEKAWI, Z. 2018. Hepatitis E virus isolated from chronic hepatitis B patients in Malaysia: Sequences analysis and genetic diversity suggest zoonotic origin. *Alexandria journal of medicine,* 54**,** 487-494.

15. JUPATTANASIN, S., CHAINUVATI, S., CHOTIYAPUTTA, W., CHANMANEE, T., SUPAPUENG, O., CHAROONRUANGRIT, U., OOTA, S. & LOUISIRIROTCHANAKUL, S. 2019. A nationwide survey of the seroprevalence of hepatitis E virus infections among blood donors in Thailand. *Viral Immunology,* 32**,** 302-307.

16. JUTAVIJITTUM, P., JIVIRIYAWAT, Y., JIVIRIYAWAT, W., YOUSUKH, A., HAYASHI, S., ITAKURA, H. & TORIYAMA, K. 2000. Seroprevalence of antibody to hepatitis E virus in voluntary blood donors in northern Thailand. *熱帯医学 Tropical medicine,* 42**,** 135-139.

17. KHOUNVISITH, V., TRITZ, S., KHENKHA, L., PHOUTANA, V., KEOSENGTHONG, A., POMMASICHAN, S., NOUANTHONG, P., HÜBSCHEN, J. M., SNOECK, C. J. & REINHARZ, D. 2018. High circulation of Hepatitis E virus in pigs and professionals exposed to pigs in Laos. *Zoonoses and public health,* 65**,** 1020-1026.

18. LORENZO, A. A., DE GUZMAN, T. S. & SU, G. L. S. 2015. Detection of IgM and IgG antibodies against hepatitis E virus in donated blood bags from a national voluntary blood bank in Metro Manila, Philippines. *Asian Pacific Journal of Tropical Disease,* 5**,** 604-605.

19. LOUISIRIROTCHANAKUL, S., MYINT, K., SRIMEE, B., KANOKSINSOMBAT, C., KHAMBOONRUANG, C., KUNSTADTER, P. & WASI, C. 2002. The prevalence of viral hepatitis among the Hmong people of northern Thailand. *Southeast Asian journal of tropical medicine and public health,* 33**,** 837-844.

20. MYINT, K. S. A., DURIPUNT, P., MAMMEN JR, M. P., SIRISOPANA, N., RODKVAMTOOK, W. & GIBBONS, R. V. 2007. Hepatitis E virus infection in Thai troops deployed with UN peacekeeping forces. *Military medicine,* 172**,** 1217-1219.

21. NG, K., HE, J., SAW, T. & LYLES, C. 2000. A seroprevalence study of viral hepatitis E infection in human immunodeficiency virus type 1 infected subjects in Malaysia. *The Medical journal of Malaysia,* 55**,** 58-64.

22. NOUHIN, J., BARENNES, H., MADEC, Y., PRAK, S., HOU, S. V., KERLEGUER, A., KIM, S., PEAN, P. & ROUET, F. 2015. Low frequency of acute hepatitis E virus (HEV) infections but high past HEV exposure in subjects from Cambodia with mild liver enzyme elevations, unexplained fever or immunodeficiency due to HIV-1 infection. *Journal of Clinical Virology,* 71**,** 22-27.

23. NOUHIN, J., MADEC, Y., PRAK, S., ORK, M., KERLEGUER, A., FROEHLICH, Y., PAVIO, N. & ROUET, F. 2019. Declining hepatitis E virus antibody prevalence in Phnom Penh, Cambodia during 1996–2017. *Epidemiology & Infection,* 147.

24. NOUHIN, J., PRAK, S., MADEC, Y., BARENNES, H., WEISSEL, R., HOK, K., PAVIO, N. & ROUET, F. 2016. Hepatitis E virus antibody prevalence, RNA frequency, and genotype among blood donors in Cambodia (Southeast Asia). *Transfusion,* 56**,** 2597-2601.

25. PILAKASIRI, C., GIBBONS, R. V., JARMAN, R. G., SUPYAPOUNG, S. & MYINT, K. S. A. 2009. Hepatitis antibody profile of Royal Thai Army nursing students. *Tropical Medicine & International Health,* 14**,** 609-611.

26. POOVORAWAN, Y., THEAMBOONLERS, A., CHUMDERMPADETSUK, S., KOMOLMIT, P. & THONG, C. 1996. Prevalence of hepatitis E virus infection in Thailand. *Annals of Tropical Medicine & Parasitology,* 90**,** 189-196.

27.SA-NGUANMOO, P., POSUWAN, N., VICHAIWATTANA, P., WUTTHIRATKOWIT, N., OWATANAPANICH, S., WASITTHANKASEM, R., THONGMEE, T., POOVORAWAN, K., THEAMBOONLERS, A. & VONGPUNSAWAD, S. 2015. Swine is a possible source of hepatitis E virus infection by comparative study of hepatitis A and E seroprevalence in Thailand. *PloS one,* 10.

28. SEDYANINGSIH-MAMAHIT, E., LARASATI, R., LARAS, K., SIDEMEN, A., SUKRI, N., SABARUDDIN, N., DIDI, S., SARAGIH, J., MYINT, K. & ENDY, T. 2002. First documented outbreak of hepatitis E virus transmission in Java, Indonesia. *Transactions of the Royal Society of Tropical Medicine and Hygiene,* 96**,** 398-404.

29. SEOW, H. F., MAHOMED, N. M. B., MAK, J. W., RIDDELL, M. A., LI, F. & ANDERSON, D. A. 1999. Seroprevalence of antibodies to hepatitis E virus in the normal blood donor population and two aboriginal communities in Malaysia. *Journal of medical virology,* 59**,** 164-168.

30. SIRIPANYAPHINYO, U., BOON‐LONG, J., LOUISIRIROTCHANAKUL, S., TAKEDA, N., CHANMANEE, T., SRIMEE, B., NAMSAI, A., POUNSAWAT, P. & KHUPULSAP, K. 2014. Occurrence of hepatitis E virus infection in acute hepatitis in Thailand. *Journal of medical virology,* 86**,** 1730-1735.

31. SURYA, I. G. P., KORNIA, K., SUWARDEWA, T. G. A., TSUDA, F. & MISHIRO, S. 2005. Serological markers of hepatitis B, C, and E viruses and human immunodeficiency virus type‐1 infections in pregnant women in Bali, Indonesia. *Journal of medical virology,* 75**,** 499-503.

32. TRAN, H. T.-T., USHIJIMA, H., QUANG, V. X., PHUONG, N., LI, T.-C., HAYASHI, S., LIEN, T. X., SATA, T. & ABE, K. 2003. Prevalence of hepatitis virus types B through E and genotypic distribution of HBV and HCV in Ho Chi Minh City, Vietnam. *Hepatology research,* 26**,** 275-280.

33. TRITZ, S. E., KHOUNVISITH, V., POMMASICHAN, S., NINNASOPHA, K., KEOSENGTHONG, A., PHOUTANA, V., CAMOIN, M., HÜBSCHEN, J. M., BLACK, A. P. & MULLER, C. P. 2018. Evidence of increased Hepatitis E virus exposure in Lao villagers with contact to ruminants. *Zoonoses and public health,* 65**,** 690-701.

34. UCHIDA, T., AYE, T. T., MA, X., IIDA, F., SHIKATA, T., ICHIKAWA, M., RIKIHISA, T. & WIN, K. M. 1993. An epidemic outbreak of hepatitis E in Yangon of Myanmar: antibody assay and animal transmission of the virus. *Pathology International,* 43**,** 94-98.

35. UTSUMI, T., HAYASHI, Y., LUSIDA, M. I., AMIN, M., HENDRA, A., YANO, Y. & HOTTA, H. 2011. Prevalence of hepatitis E virus among swine and humans in two different ethnic communities in Indonesia. *Archives of virology,* 156**,** 689-693.

36. WIBAWA, I. D. N., MULJONO, D. H., SURYADARMA, I., TSUDA, F., TAKAHASHI, M., NISHIZAWA, T. & OKAMOTO, H. 2004. Prevalence of antibodies to hepatitis E virus among apparently healthy humans and pigs in Bali, Indonesia: Identification of a pig infected with a genotype 4 hepatitis E virus. *Journal of medical virology,* 73**,** 38-44.

37. WIBAWA, I. D. N., SURYADARMA, I., TSUDA, F., MATSUMOTO, Y., NINOMIYA, M., TAKAHASHI, M. & OKAMOTO, H. 2007. Identification of genotype 4 hepatitis E virus strains from a patient with acute hepatitis E and farm pigs in Bali, Indonesia. *Journal of medical virology,* 79**,** 1138-1146.

38. WIDASARI, D. I., YANO, Y., UTSUMI, T., HERIYANTO, D. S., ANGGOROWATI, N., RINONCE, H. T., UTORO, T., LUSIDA, M. I., ASMARA, W. & HOTTA, H. 2013. Hepatitis E virus infection in two different regions of Indonesia with identification of swine HEV genotype 3. *Microbiology and immunology,* 57**,** 692-703.

39. WONG, C. C., THEAN, S. M., NG, Y., KANG, J. S. L., NG, T. Y., CHAU, M. L., KOH, T. H. & CHAN, K. P. 2019. Seroepidemiology and genotyping of hepatitis E virus in Singapore reveal rise in number of cases and similarity of human strains to those detected in pig livers. *Zoonoses and public health,* 66**,** 773-782.

40. WONG, L. P., ALIAS, H., CHOY, S. H., GOH, X. T., LEE, S. C., LIM, Y. A. L., KEE, B. P., CHUA, K. H., KAMARUZAMAN, A. & ZHENG, Z. 2020. The study of seroprevalence of hepatitis E virus and an investigation into the lifestyle behaviours of the aborigines in Malaysia. *Zoonoses and Public Health,* 67**,** 263-270.

41. YAMADA, H., TAKAHASHI, K., LIM, O., SVAY, S., CHUON, C., HOK, S., DO, S. H., FUJIMOTO, M., AKITA, T. & GOTO, N. 2015. Hepatitis E virus in Cambodia: Prevalence among the general population and complete genome sequence of genotype 4. *PLoS One,* 10**,** e0136903.
